# Intelligent Diagnosis and Analysis of Brain Lymphoma Based on DSC Imaging Features

**DOI:** 10.1155/2022/4981620

**Published:** 2022-02-24

**Authors:** Yipu Mao, Muliang Jiang, Fanyu Zhao, Liling Long

**Affiliations:** ^1^Department of Radiology, The First Affiliated Hospital of Guangxi Medical University, Nanning 530021, Guangxi, China; ^2^Department of Radiology, Guangxi Zhuang Autonomous Region Ethnic Hospital, Nanning 530021, Guangxi, China

## Abstract

Currently, DSC has been extensively studied in the diagnosis, differential diagnosis, and prognosis evaluation of brain lymphoma, but it has not obtained a uniform standard. By combining DSC imaging features, this study investigated the imaging features and diagnostic value of several types of tumors such as primary brain lymphoma. At the same time, this study obtained data from brain lymphoma patients by data collection and set up different groups to conduct experimental studies to explore the correlation between IVIMMRI perfusion parameters and DSC perfusion parameters in brain lymphoma. Through experimental research, it can be seen that the combination of two perfusion imaging techniques can more fully reflect the blood flow properties of the lesion, which is beneficial to determine the nature of the lesion.

## 1. Introduction

The improvement of diagnostic accuracy and improvement of prognosis in patients with PCNSL has been one of the hotspots of scholars at home and abroad. Congenital and acquired immunodeficiency is currently the only identified risk factor for the disease. In particular, people living with HIV have a greater risk of developing the disease than the normal population. According to the research of the past decade, it is found that although the incidence of PCNSL is increasing year by year, the incidence of immunodeficiency is reduced. The reason is that the high-efficiency antiretroviral therapy (HAART) is widely used in the clinical treatment of AIDS, which reduces the prevalence of PCNSL in AIDS patients [1]. There are also reports that with the continuous development of cancer treatment technology, compared with the past, the survival rate of PCNSL patients, especially younger patients, has been significantly improved [2]. Whether it is an immune-sufficient population or an immunodeficient population, if it can be diagnosed early, it will play an important role in guiding the clinical effective treatment plan to improve patient survival and improve prognosis. Magnetic resonance imaging has a high sensitivity in the localization and characterization of intracranial tumors, and it has great significance in the diagnosis, differential diagnosis, prognosis evaluation, and evaluation of treatment plans of PCNSL. The imaging principle, characteristics, current status, and development prospects of traditional magnetic resonance imaging and various emerging magnetic resonance techniques in the diagnosis of PCNSL are reviewed.

In the MRI routine examination, the researchers observed that PCNSL generally occurs on the screen, and it mainly appears in the white matter of the brain and the side of the lateral ventricles, such as the thalamus, corpus callosum, and basal ganglia. Kulker et al. reported that in immunocompetent PCNSL patients, single-shot lesions accounted for 65%, lesions in the cerebral hemisphere accounted for 38%, thalamic/basal ganglia lesions accounted for 16%, and corpus callosum lesions accounted for 14%, and around the ventricles, the lesions in the area accounted for 12%, the lesions in the cerebellum were about 9%, and it rarely involved the dura mater. PCNSL can be expressed as a single lesion or multiple lesions in patients with immune function [3].

MRI plain scan of PCNSL generally showed TIWI lesions showed equal or slightly lower signal; T2WI showed equal or slightly higher signal but showed equal or low signal shadow relative to normal brain gray matter; lesions of calcification or hemorrhage are rare, and edema around the lesion is more severe than other intracranial tumors, but the mass effect is more severe [4]. These basic imaging findings can make PCNSL relatively specific, but, in some cases, T2WI appears to be a normal signal. Some investigators have performed an autopsy on these patients with a special performance and believe that this may be due to the slight infiltration of the surrounding tissue by the tumor. Therefore, the negative MR performance cannot completely rule out the diagnosis of PCNSL [5].

By MRI-enhanced scanning, the lesions were significantly and evenly enhanced. Some scholars believe that this is because PCNSL has strong invasiveness, which destroys the blood-brain barrier and causes leakage of contrast agents. The degree of enhancement is related to the degree of damage of the blood-brain barrier, and the way of strengthening may depend on the autoimmune function of PCNSL patients. A large number of case studies at home and abroad have shown that patients with immunodeficiency in PCNSL have more common tumor cystic necrosis, and lesion enhancement usually appears as “ring”. However, for patients with immune-sound PCNSL, lesions and necrosis are rare, and the enhancement usually presents as a typical “horse hoof”, “butterfly sign”, or “clump” [6]. Immunodeficient PCNSL has the property of invading the ependymal membrane, which can be fully displayed on enhanced T1WI. A large number of case studies have shown that conventional MRI is of great value in the diagnosis of PCNSL. However, the imaging performance of PCNSL is often unstable in conventional MR examination, and it lacks characteristic imaging findings, and these shortcomings will become the direction of future research. At present, conventional MRI evaluates the response of PCNSL to radiotherapy and chemotherapy according to the change in lesion size on T1WI after treatment [7]. In recent years, some scholars have found that after a large dose of methotrexate chemotherapy, if the MRI-enhanced scan lesions are evenly enhanced, then patients with PCNSL have a better prognosis, but the prognosis of patients with intensified intensification is generally poor [8]. Some scholars have found that after PCNSL patients undergo chemotherapy and MRI scan, if the TlWl signal of the lesion increases, it indicates that the tumor cells respond to the treatment. Therefore, in future research, the value of conventional MRI examination in evaluating various treatment effects of PCNSL can be further explored.

Perfusion weighted imaging (PWI) is mainly used for the study of microscopic hemodynamics. The growth pattern of tumor nourishing blood vessels has always been a hot research direction for scholars at home and abroad. The emergence of PWI technology provides a visual research method. The pathological process of PCNSL determines the growth rate and structural specificity of the tumor nourishing blood vessels, and the degree of malignancy of PCNSL is high, which easily destroys the blood-brain barrier, so the lesion area must have obvious blood flow changes. PWI examination revealed that although the rrCBV of PCNSL was elevated, it was much lower than that of high-grade astrocytoma and meningioma. The enhancement of PCNSL only reflects the degree of damage of the blood-brain barrier and does not reflect the degree of tumor angiogenesis. Because PCNSL lesion itself has no obvious blood supply, PWI showed a low perfusion mass, cerebral blood volume and cerebral blood flow decreased, average passage time and peak time prolonged, which was significantly different from high perfusion of high-grade glioma [8]. In recent years, studies have shown that CBV values without contrast-deficient correction are the best indicators for identifying PCNSL and gliomas by PWI. Some scholars have applied PWI to the study of the ischemic penumbra around the PCNSL lesion and observed a slight increase in rrCBV in the penumbra [9]. The numerous parameter indicators and comparison methods of PWI examination provide a new and effective means for clinical diagnosis and evaluation of PCNSL.

Magnetic resonance spectroscopy (MRS) is the only noninvasive technique for determining the chemical composition of a particular tissue region in a living body and provides metabolic information for the tissue. The molecular structure of different metabolites is different, the chemical shifts of protons in the molecule are different, and the precession frequency is also different. The MRS signal generation is based on this principle [10]. The MRS characteristic of PCNSL is characterized by a moderate decrease in NAA, an increase in Cho, a decrease in Cr, and an increase in Cho/Cr and Cho/NAA [11].

Although pleomorphic gliomas also have the above-mentioned performance, some scholars have found that the Lip peak of PCNSL is much higher than that of various types of glioma. This spectroscopy is explained by the fact that the rise of the Lip peak of glioma is due to cell necrosis, while the PCNSL is due to the massive accumulation of macrophages in the tumor and the metabolism of the apoptotic lymphocyte membrane. At present, with the establishment of computer programs and mathematical models, the interest of scholars in various countries in MRS research has shifted from qualitative research on metabolites to quantitative research. This transformation provides new insights into the identification of PCNSL and other intracranial tumors, as well as the basis for imaging to provide a metabolic basis for evaluating PCNSL activity and therapeutic effects.

## 2. Research Method

### 2.1. Research Object

DSC-MR perfusion imaging is the most commonly used perfusion method in exogenous tracer imaging. In the field of MR perfusion imaging, its advantages are high signal-to-noise ratio and high time resolution, so it has become a more common and mature MR perfusion imaging technology. It mainly reflects the permeability and blood perfusion of the microcirculation of the diseased tissue after contrast agent injection; that is, it reflects the degree of new capillary, vascular permeability, and maturity of the lesion. Compared to the “generalization” of conventional MRI-enhanced scanning, its “thinning” functional imaging principle can obtain more enhanced scanning parameter information.

A total of 65 patients with brain tumors underwent double-exponential model fitting of IVIMMRI, and brain dynamic magnetic contrast-enhanced scans were performed between November 2017 and November 2018.3 patients without pathology were excluded, 2 patients were tumor recurrence, 6 patients were low-grade glioma (pathological diagnosis of DNT), and 3 patients were cavernous hemangioma. Therefore, a total of 51 patients were enrolled. Before the magnetic resonance examination, there were 20 cases of high-grade astrocytoma (14 cases of glioblastoma and 6 cases of deformed astrocytoma) confirmed by stereotactic biopsy or surgical pathology, 20 cases of meningioma, and 11 cases of primary brain lymphoma. Patient exclusion criteria were included as follows: 1. patients with severe cachexia symptoms who are unable to cooperate with the examination, 2. patients with renal insufficiency who are unable to receive intravenous contrast injection, 3. patients who have undergone pathological puncture, 4. patients who have been treated (including steroids, surgery, radiotherapy, and chemotherapy), 5. minors, 6. patients with contraindications for MR examination such as cardiac pacemakers, cochlear implants, patients after plate implantation, patients with metal dentures, patients with claustrophobia, or patients with poor compliance, and 7. patients with recurrent tumors. There were 10 cases of lymphoma confirmed by stereotactic biopsy. There were 1 case of primary brain lymphoma, 20 cases of high-grade astrocytoma (14 cases of glioblastoma and 6 cases of interstitial astrocytoma), and 20 cases of meningioma confirmed by operation and pathology. Twenty patients with high-grade astrocytoma and 20 patients with meningioma were treated with surgical resection of the tumor. Eleven patients with brain lymphoma were transferred to our hospital for lymphatics for chemotherapy (first-line chemotherapy regimens were methotrexate, temozolomide, and rituximab) [12].

### 2.2. Research Methods

The Philips Achieva 1.5 T and the Siemens Skyra-FREEDOM 3.0 T were used as experimental instruments, the standard head quadrature coil was applied, and the patient was placed in a supine position. All experimental groups underwent routine head MR plain scan and DWI scan, and then, a dynamic magnetic sensitivity contrast-enhanced MR imaging (DSC-MRI) scan was performed [13].

MRI examination①: plain sweep echo sequence (SE) T1WI (axis position): repetition time (TR) is 380 ms, echo time (TE) is 13.0 ms, layer thickness is 6 mm, and FOV 220 × 176 mm, matrix 320 × 288. T2WI (axis position): repetition time (TR) is 3100 ms, echo time (TE) is 93.0 ms, inversion time (TI) is 2500 ms, layer thickness is 6 mm, FOV 220 × 176 mm, matrix 320 × 256. MRI②DSC scan: A weighted single-shot gradient echo plane echo imaging (GREEPI) sequence was used. The scanning parameters are TR1410 ms and TE30 ms, flip angle is 60, the number of excitations is 1, the field of view (FOV) is 24 cm × 24 cm, the matrix is 128 x 128, the layer thickness is 5 mm, the layer spacing is 1.5 mm, the number of layers is 19 layers, and the acquisition time is 1 min 18 s. The contrast agent was Gd-DTPA, which was injected through the elbow vein at a flow rate of 5 ml/s using an MR high-pressure syringe at a dose of 0.1 mmol/kg body weight, and then, the same amount of physiological saline was injected at the same flow rate [14].

The original PWI image was analyzed and processed by Siemens Skyra-FREEDOM 3.0 T, and MRI diagnosticians with more than 5 years of experience were invited to observe the abnormal perfusion area. The experimental group selected the maximal level of abnormal perfusion of the lesion and loaded the original map of the plane into Skyra-FREEDOM 3.0 T. The region of interest (ROI) was placed in the perfusion abnormal zone to obtain the time-signal intensity curve of the lesion. The placement of ROI should avoid the influence of peripheral blood vessels and hemorrhage. The time-signal intensity curve of the normal white matter region can be obtained by mirroring the contralateral normal white matter as the control area. The health group also manually determines the size of the ROI on the right side by manual method and then places the ROI on the opposite side by mirroring method to obtain the time-signal intensity curve of the healthy group. The intensity of each phase signal before the first pass of the contrast agent is used as the baseline signal, and the signal intensity of each phase after the first pass of the contrast agent is used as the recovery signal. Moreover, the peak value of the curve (ΔH) is the absolute value of the difference between the maximum values of the signal intensity changes of the brain tissue during the first pass of the contrast agent, and the time difference (ΔT) is the time difference between the peaks of the brain tissue on the time-signal intensity curve [15].

Automatically, cerebral blood flow (CBF), cerebral blood volume (CBV), mean transit time (MTT), and maximum peak time (TTP) pseudo-color maps were acquired. The region of interest is determined by the artificial method (the region of interest is consistent with the region of interest in the time-signal intensity curve) and placed in the lesion area of the CBF, CBV, MTT, TTP parameter map, the periphery of the lesion, and the blood supply area of the responsible vessel. The ROI was placed on the opposite side by the mirror method, and the average value was taken to calculate the ratio of the affected side divided by the healthy side. The health group also manually determined the size of the region of interest (ROI) on the right side by manual method and then placed it on the opposite side by the mirror method ROI, and the ratio of the two sides was calculated (right/left side) [16].

The extent of abnormal enhancement of glioma patients after surgery and radiotherapy after chemotherapy was determined by two high-ranking radiologists. Moreover, on the perfusion imaging images (DSC, DCE) corresponding to the maximum level within the enhancement range of the conventional axial T1WI-weighted enhancement display, the corresponding regions of interest are separately outlined (the regions of interest, ROI), the relationship of all regions of interest is changed over time, and the average area of the fixed ROI is 10 mm^2^. The parenchymal area of the tumor is mainly measured, and the blood vessels, hemorrhage, cystic changes, and necrotic areas are avoided. The ROI of each lesion was measured 5 times in ROI, and the dynamic contrast-enhanced magnetic resonance imaging was applied with kinetic model mg. version 3.0 permeation analysis software. Moreover, the contralateral normal middle cerebral artery was selected as the arterial input function (toe F), and the extended Tofts model hemodynamic model was used to obtain the time and signal curve. The DCE hemodynamic parameters obtained by calculation include Ktrans, Ve, kep, Vp, riAUC, and functional pseudo-color maps corresponding to each parameter. Meanwhile, DSC uses Siemens Perfusion postprocessing software to obtain relevant parameter values including rCBV, rCBF (lesion area/contralateral normal brain tissue), and functional pseudo-color map corresponding to each parameter in the corresponding ROI [17].

## 3. Result

In the DWI map, 42 lesions showed high signal and 5 lesions had DWI contour signals. The ADC values were lower than those of the contralateral normal white matter region.

**Figure 1:**
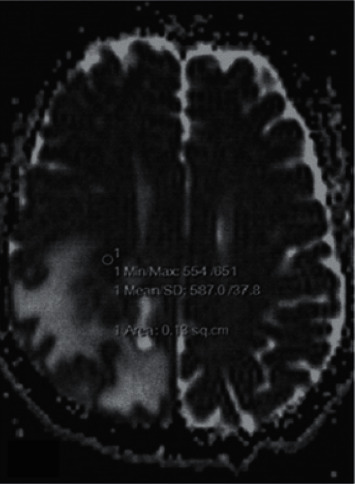
The ADC image of the corresponding level and the lesion enhancement part show a low signal in the ADC map.

**Figure 2:**
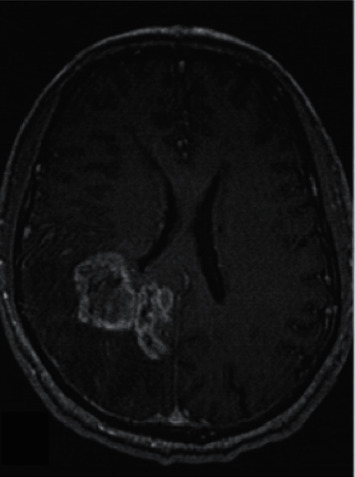
GBM, axial position enhancement, significant enhancement of the parenchymal part of the right occipital lobe.

rADC<1 was 100%, and the lesions were significantly enhanced after enhancement. In high-grade gliomas, 35 lesions showed high signal on DWI, 3 lesions showed slightly higher signal, and ADC values were lower than those in contralateral normal white matter region (see Figure 1). After enhancement, the parenchymal part of the lesion was significantly enhanced (seeing Figure 2). However, there was no significant high signal on the DWI of the other 4 lesions, and the ADC value of the parenchymal part of the lesion was lower than that of the contralateral normal white matter region.

**Figure 3:**
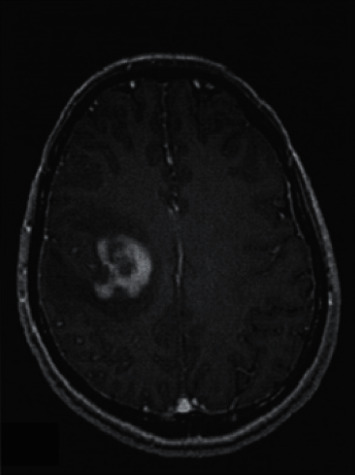
Inflammatory demyelination, axial enhancement, and significant enhancement and alteration of the right temporal lobe lesion.

**Figure 4:**
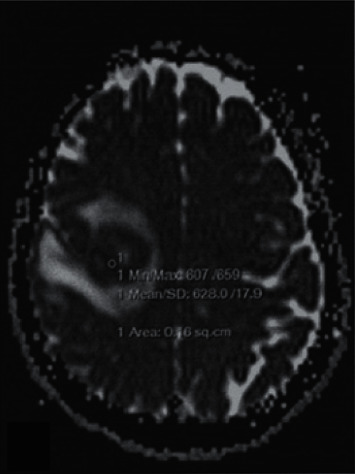
Enhanced part of the lesion appears as a low signal.

In the inflammatory demyelination, 7 lesions showed higher signal on the DWI map, and 5 lesions showed equal signals, and the ADC values were lower than those in the contralateral normal white matter region (seeing Figure 3). Moreover, the lesions were significantly enhanced after enhancement (seeing Figure 4). The following table shows the rADC situation in PCNSL, OBM, and inflammatory demyelination (seeing Table 1).

In 9 cases, the peak of the dynamic curve of the affected side was significantly delayed, and the peak was lower than the contralateral side. The overall level of the curve was lower than the contralateral side (Figure 2). ΔH was 83.23 ± 39.46 (*x* ± *s*), and ΔT was 0.05 ± 0.03. After *t*-test, the difference of △H, △T between the brain and the control group was found to be very significant (*P* < 0.01). Perfusion parameters showed asymmetry of bilateral cerebral hemisphere perfusion, decreased CBF and CBV, and increased MTT and TTP. The ratio of the focal side to the contralateral side was 0.36 ± 0.15, 0.51 ± 0.24, 1.42 ± 0.30, and 1.34 ± 0.27 on CBF, CBV, MTT, and TTP, respectively (seeing Table 2).

**Figure 5:**
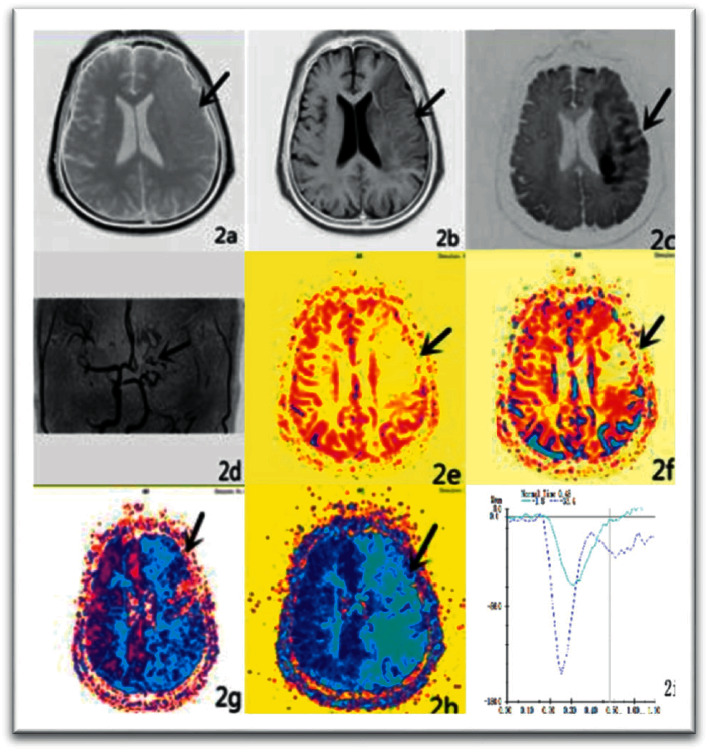
Delayed low perfusion group.

As shown in Figure 5, the patient complained that “the right limb was weak for 10 hours” and was admitted to the hospital. Conventional MRI showed a large-scale abnormal signal in the left frontal temporal lobe, T1WI (a) showed low signal, T2WI (b) and DWI (c) showed high signal, and the boundary was not clear. MRA (d) showed occlusion of the left middle cerebral artery, and its distal branch decreased, CBF (e) and CBV (f) decreased, and MTT(g) and TTP (h) increased. The time-signal intensity curve (red solid line for the cook side and yellow dashed line for the opposite side) is delayed low perfusion. The dark color in the pseudo-color map represents the corresponding perfusion parameter value increase, and the light color represents the corresponding perfusion parameter value reduction.

**Figure 6:**
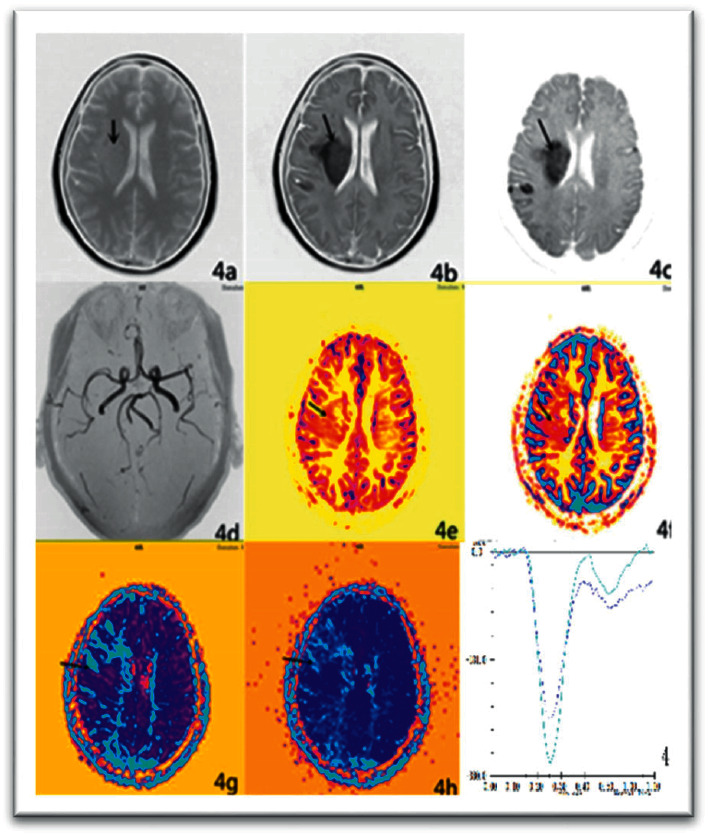
High perfusion group.

In 15 cases, the peak of the dynamic curve of the affected side was higher than that of the contralateral side, but there was no significant time delay, and the overall level of the curve was higher than that of the contralateral side (Figure 6) △H is 82.02 ± 36.70 (*x* ± *s*), and ΔT is 0.01 ± 0.01. After *t*-test, the difference of △H between the lateral brain tissue and the healthy group was found to be very significant (*P* < 0.01), and the difference between △T and the healthy group was not statistically significant (*P* > 0.05). Perfusion parameters showed that the perfusion of the bilateral cerebral hemispheres was asymmetrical, and the CBF and CBV on the lesion side increased, and the MTT and TTP increased slightly; the ratio of the lesion side to the contralateral side was 1.88 ± 0.74, 1.58 ± 0.21, 1.20 ± 0.20, and 1.12 ± 0.07 on CBF, CBV, MTT, and TTP, respectively (see Table 3).

Conventional MRI showed that the left frontal temporal lobe showed a large lamella abnormal signal, T1WI (a) showed low signal, T2WI (b) and DWI (c) showed high signal, the border was unclear, and MRA (d) showed left middle cerebral artery occlusion, and its distal branch decreased. The local areas of the lesions increased CBF (e) and CBV (f), MTT(g) and TTP (h) increased slightly, and the time-signal intensity curve showed high perfusion (the solid red line is the cooker side, and the yellow dotted line is the opposite side.). The dark color in the pseudo-color map represents an increase in the corresponding perfusion parameter value, and the light color represents a decrease in the corresponding perfusion parameter value.

To further analyze the differences in hemodynamic parameters between the four different perfusion types of cerebral infarction and the healthy group, the comparison between the experimental groups and the control group was taken, namely Dunnett's t-test. The results of each perfusion group parameter compared with the health group are as follows: (1) in the delayed perfusion group, the ratio of the lesion side to the contralateral side, the ratio of the peripheral of the lesion side to the peripheral of the contralateral side, and the ratio of the lesion side responsible for vascular blood supply area to the contralateral side responsible for vascular blood supply area were statistically significant in CBV, CBF, MTT, and TTP. (2) The ratio of lesion side to the contralateral side in the low perfusion group was statistically significant on CBV and CBF, and there was no statistical significance in MTT and TTP. The ratio of the peripheral of the lesion side to the peripheral of the contralateral side and the ratio of the lesion side responsible for vascular blood supply area to the contralateral side responsible for vascular blood supply area were not statistically significant on CBV, CBF, MTT, and TTP. (3) The ratio of lesion side to the contralateral side in the high perfusion group was statistically significant on CBV, CBF, MTT, and TTP. The ratio of the peripheral of the lesion side to the peripheral of the contralateral side and the ratio of the lesion side responsible for vascular blood supply area to the contralateral side responsible for vascular blood supply area were not statistically significant on CBV, CBF, MTT, and TTP. (4) In the irregular group, the ratio of lesion side to the contralateral side, the ratio of the peripheral of the lesion side to the peripheral of the contralateral side, and the ratio of the lesion side responsible for vascular blood supply area to the contralateral side responsible for vascular blood supply area were statistically significant in CBV, CBF, MTT, and TTP [18].

## 4. Analysis and Discussion

In recent years, through the summary analysis of the imaging performance of a large number of PCNSL cases, the imaging diagnosis has more research, and the main means also rely on MRI. In addition to plain and enhanced MRI, there are also inspection sequences such as DWI, DTIPWI, SWI, and MRS, each of which has its own unique characteristics. The performance of PCNSL in each of these sequences is characterized by a comprehensive interpretation of several sequences, which can establish a diagnosis and distinguish it from other diseases. In recent years, DWI, DTI, PWI, SWI, and MRS have made great progress as new research directions. Through the retrospective analysis of multiple confirmed pathologies, the performance characteristics of the above sequences have been preliminarily obtained, which has helped the diagnosis and differential diagnosis and improved the accuracy. However, it should be noted that the imaging performance of PCNSL is not very stable, and it is similar to other kinds of intracranial tumors, and the number of cases is small, which increases the difficulty of distinguishing it from other tumors in the brain. For prognostic assessment, there is a lack of effective observations.

By combining DCE optimal quantitative parameter Ktrans and DSC optimal semiquantitative parameter rCBV, glioma recurrence or radiation brain damage is diagnosed, In the parallel test, when the Ktrans value is higher than 1.47 minJ or the rCBV is higher than 1.861, the sensitivity for diagnosing glioma recurrence is 72.7%, and the specificity is 100%. In the tandem test, when the Ktrans value is higher than 1.47 minJ or the rCBV is higher than 1.861, the sensitivity for diagnosing glioma recurrence is 90.9%, and the specificity is 75%. The combined diagnosis of hemodynamic parameters Ktrans and rCBV can improve the differential diagnosis efficiency [19].

Dynamic magnetic-sensitive contrast-enhanced imaging is the most common perfusion imaging method, and some studies have confirmed that DSC-MXI has important diagnostic value in distinguishing glioma recurrence or radioactive necrosis. The reason is that the cell metabolism is extremely vigorous when the tumor recurs, the release of vascular endothelial growth factor is stimulated, the number of blood vessels is rapidly increased, the diameter of the tube is increased, and the blood vessel density is increased. Moreover, the damage of brain tissue caused by radiation has no neovascularization, which often leads to damage or necrosis of vascular endothelial cells, a slight increase in permeability, and a decrease in blood perfusion compared with the contralateral side. Therefore, the nature of the lesion can be analyzed by detecting the difference in blood flow, blood volume, and normal brain tissue in the diseased blood vessel. The tumor recurrence was higher than the pseudo-progression or radioactive necrosis, and the diagnostic efficiency of rCBF was lower than that of rCBV, which was almost the same as that of Sugahara. It is believed that rCBV is the most important parameter for the diagnosis of high-grade glioma recurrence and pseudo-progression or radioactive necrosis after radiotherapy. MRI is a single-chamber hemodynamic model based on the intact blood-brain barrier. It does not take into account that high-grade gliomas may disrupt the blood-brain barrier and cause some contrast agents to leak out of the blood vessels and cause an underestimation of rCBV and rCBF, and there is a lack of a linear quantitative relationship between the contrast agent and the signal. Moreover, the GER-EPI sequence used is susceptible to magnetically sensitive artifacts from large blood vessels and bones, which leads to poor lesions at the base of the skull [20].

All patients in this study underwent re-examination of magnetic resonance within two weeks after surgery, and radiotherapy and chemotherapy were started three weeks after surgery to eliminate and avoid the inflammatory response enhancement of the residual cavity after the operation, which affected the determination of the range of abnormal enhancement lesions during the follow-up of the operation area. At the same time, this study innovatively obtained the time-signal intensity curve of two groups of perfusion imaging and the hemodynamic parameters related to perfusion imaging by one injection of contrast agent. In addition, this study explored and compared the clinical value of these two noninvasive perfusion imaging in the identification of glioma recurrence or radiation-induced brain injury. Previous studies on this problem have focused on the application of a single new technology or a combination of DWI, DTI, etc., and there are few studies combining two perfusion imaging. By comparing the perfusion imaging parameters and the final pathology or follow-up results, it showed that the rCBV, rCBF, Ktrans, Ve, and riAUC of the radiation-induced brain injury group were lower than those of the glioma recurrence group. The diagnostic power of rCBV in DSC alone is slightly higher than that of Ktrans. If it is combined with Ktrans and rCBV in parallel diagnosis, the sensitivity of differential diagnosis is 72.7%, and the specificity is 100%. If it is combined with Ktrans and rCBV in tandem diagnosis, the sensitivity can be increased to 90.9%, and the specificity is 75%. Both DSC and DCE can be used to identify glioma recurrence or radiation damage, and the combined determination of the two perfusion methods can improve the diagnostic efficacy. DSC mainly evaluates the relative cerebral blood volume and cerebral blood flow of tumor-side blood vessels under the condition that the vascular barrier is not damaged, and DCE mainly evaluates the permeability of new blood vessels. It can be seen that the combination of the two perfusion imaging techniques can more fully reflect the blood flow properties of the lesion, thereby facilitating the determination of the nature of the lesion.

## 5. Conclusion

There is little difference in the diagnostic efficacy of DCE and DSC hemodynamic parameters in identifying tumor recurrence or radiation-induced brain injury. The diagnostic efficacy of Ktrans, riAUC, and rCBV is slightly higher than Ve and rCBF, and the combined diagnosis of rCBV and Ktrans can improve the diagnostic accuracy. Both DCE and DSC can be used to guide clinicians to distinguish glioma recurrence or radiation brain damage at an early stage, so as to guide clinicians to the correct treatment when abnormal augmentation is found. In this study, two sets of blood flow perfusion analysis parameters were obtained by one injection of contrast agent to distinguish tumor recurrence or injury. Moreover, this experiment compared the differences in the important imaging parameters of the two techniques in the differential diagnosis and the value and significance of the joint diagnosis, and there have been few studies in the past. However, there are some limitations in this study, and the artificial delineation of the region of interest in postprocessing does not take into account the difference in tumor heterogeneity will produce selective bias, and the hemodynamics of DCE is complex, and the types of postprocessing models are many and nonuniform, which leads to poor comparability between the research results, and the diagnostic threshold cannot be accurately defined. Therefore, this new technology is currently limited in clinical application.

## Figures and Tables

**Table 1:** Present quantity, mean, standard deviation, standard error, 95% confidence interval, minimum and maximum value.

Group	rADC value	Confidence interval	Maximum value	Minimum value
Lymphoma	0.68 + 0.12	(0.64, 0.71)	0.43	0.9
Glioblastoma	0.77 + 0.60	(0.75, 0.78)	0.67	0.9
Inflammatory demyelination	0.83 + 0.0.07	(0.79, 0.88)	0.75	0.94

**Table 2:** Ratio of blood perfusion parameters in the delayed hypoperfusion group.

Parameter	Cooker side/contralateral side	Lesion side/contralateral side	Lesion side area that responsible for vascular blood supply area/contralateral area that responsible for vascular blood supply area
rCBF	0.36 ± 0.15	0.49 ± 0.13	0.48 ± 0.14
rCBV	0.51 ± 0.24	0.59 ± 0.19	0.63 ± 0.16
rMTT	1.42 ± 0.30	1.41 ± 0.44	1.24 ± 0.27
rTTP	1.34 ± 0.27	1.32 ± 0.41	1.34 ± 0.43

**Table 3:** Ratio of blood perfusion parameters in the high perfusion group.

Parameter	Cooker side/contralateral side	Lesion side/contralateral side	Lesion side area that responsible for vascular blood supply area/contralateral area that responsible for a vascular blood supply area
rCBF	1.88 ± 0.74	1.19 ± 0.50	1.03 ± 0.19
rCBV	1.58 ± 0.21	1.09 ± 0.29	1.05 ± 0.17
rMTT	1.20 ± 0.20	1.01 ± 0.08	1.03 ± 0.08
rTTP	1.12 ± 0.07	1.01 ± 0.03	1.02 ± 0.03

## Data Availability

The data used to support the findings of this study are available from the corresponding author upon request.
